# Cardiovascular Magnetic Resonance for Early Detection of Cancer Therapy-Related Cardiac Dysfunction: From Multiparametric Tissue Signatures to Clinical Pathways

**DOI:** 10.3390/cancers18203342

**Published:** 2026-10-16

**Authors:** Qikai Zong, Cheng Sun, Tianxiang Gu, Enyi Shi, Shen Qu

**Affiliations:** The First Affiliated Hospital of China Medical University, Shenyang 110001, China; zongqk@foxmail.com (Q.Z.); 19991034@cmu.edu.cn (E.S.);

**Keywords:** cardio-oncology, cancer therapy-related cardiac dysfunction, cardiovascular magnetic resonance, myocardial strain, T1 mapping, immune checkpoint inhibitor myocarditis, extracellular volume fraction, early diagnosis, cancer survivorship

## Abstract

Cancer therapy-related cardiac dysfunction (CTRCD) can compromise cardiovascular health and the delivery of anticancer treatment. Echocardiography and cardiac biomarkers remain the first-line surveillance tools; cardiovascular magnetic resonance (CMR) provides complementary assessment of ventricular function and myocardial tissue when findings are inconclusive or additional characterization may change management. This narrative review examines multiparametric CMR across seven anticancer treatment groups and proposes a risk-adapted approach to its clinical use. Cine imaging, T1 and T2 mapping, extracellular volume fraction (ECV), late gadolinium enhancement, and feature-tracking strain provide different, partly overlapping information. Serial interpretation requires attention to local reference ranges, measurement reproducibility, hematocrit, and acquisition and analysis consistency. An absolute ECV increase of at least 3 percentage points is discussed as an observational risk signal, not proof of collagen deposition or a validated treatment threshold. The guideline threshold of a greater than 15% relative decline in global longitudinal strain derives from echocardiography and should not be assumed to apply directly to CMR feature tracking. We distinguish guideline-supported practice, randomized evidence, observational evidence, and mechanistic extrapolation from the authors’ proposed surveillance and rechallenge framework. Suspected immune checkpoint inhibitor myocarditis requires urgent clinical evaluation even when CMR is negative. Prospective trials are needed to establish whether CMR-informed management improves cardiovascular outcomes, treatment completion, or survival.

## Simple Summary

Some cancer treatments can damage the heart, but stopping an effective treatment unnecessarily can also harm patients. Heart ultrasound and blood tests remain the main monitoring tools. Cardiac magnetic resonance imaging can provide additional information when these tests are unclear or when the type of heart injury needs to be established. This review examines its role across seven groups of cancer treatments and discusses how imaging findings may inform heart-protective treatment and decisions about continuing cancer therapy. We distinguish recommendations supported by clinical guidelines from observational findings and proposed monitoring strategies. Particular attention is given to measurement reliability, the limitations of tissue markers, and the urgent assessment of suspected treatment-related myocarditis. Studies are still needed to determine whether the proposed imaging approach improves heart health, cancer treatment completion, or survival.

## 1. Introduction

Cardiovascular disease is an important cause of morbidity and mortality among cancer survivors [1]. Cancer therapy-related cardiac dysfunction (CTRCD) can also disrupt effective anticancer treatment. Lower relative dose intensity (RDI) has been associated with poorer cancer outcomes in several settings, including breast cancer, although the effect depends on the regimen and disease context [2,3]. Cardiac surveillance should therefore support both timely treatment of cardiovascular toxicity and appropriate continuation of cancer therapy. Cardiovascular magnetic resonance (CMR) may contribute when the nature or severity of suspected injury remains uncertain; a survival benefit from CMR-guided management has not been established.

Routine surveillance combines clinical assessment, echocardiographic left ventricular ejection fraction (LVEF) and global longitudinal strain (GLS), and cardiac biomarkers according to treatment and baseline risk. Acoustic windows and measurement variability can limit echocardiography, particularly after thoracic surgery or radiotherapy [4,5]. High-sensitivity cardiac troponin I (hs-cTnI) detects myocardial injury but does not establish its mechanism or reversibility. Cardiac CARE enrolled 175 patients receiving anthracyclines for breast cancer or lymphoma; 57 with higher on-treatment hs-cTnI concentrations were randomized to carvedilol plus candesartan or standard care [6]. The adjusted between-group difference in 6-month CMR-LVEF change was −0.37 percentage points (95% CI, −3.59 to 2.85; *p* = 0.82). At least one adverse event was reported in 71.4% versus 10.3%, respectively, and one standard-care participant developed heart failure [6]. The small randomized sample and the troponin-based selection strategy limit interpretation, as discussed in subsequent correspondence [7]. The result does not establish that troponin elevation is benign or that CMR tissue confirmation improves outcomes.

CMR provides reproducible ventricular volumes and ejection fraction without dependence on acoustic windows, together with T1 and T2 mapping, extracellular volume fraction (ECV), late gadolinium enhancement (LGE), and feature-tracking strain [8,9]. These measurements can characterize myocardial abnormalities that are not explained by LVEF alone [10]. Much of the cardio-oncology evidence concerns anthracyclines, as summarized in recent reviews [11,12]. Less well established are treatment-specific imaging windows, transferable thresholds for tissue change, and the benefit of using these findings to modify cancer treatment. This review considers these questions across therapies with different mechanisms and clinical risks.

We first review the biological rationale for early detection and the interpretation of CMR parameters (Section 2 and Section 3). Section 4 summarizes evidence for anthracyclines, anti-HER2 antibodies, vascular endothelial growth factor (VEGF)-pathway inhibitors, immune checkpoint inhibitors (ICIs), chest radiotherapy, chimeric antigen receptor T-cell (CAR-T) therapy, and proteasome inhibitors. Section 5 and Section 6 propose how CMR might complement established surveillance and multidisciplinary treatment decisions. Evidence categories are defined in Section Literature Search and Framework Development. Throughout, we distinguish findings that support reassessment or cardioprotection from those that contribute to a decision to interrupt or restart anticancer therapy.

### Literature Search and Framework Development

This narrative review draws on English-language literature identified through PubMed and cross-checked against publisher records and trial registries through September 2026. Search terms combined CMR techniques (cardiovascular magnetic resonance, T1/T2 mapping, extracellular volume, feature-tracking strain, and late gadolinium enhancement) with cardiotoxicity, CTRCD, immune checkpoint inhibitor myocarditis, and the seven treatment groups discussed in Section 4. Guidelines, primary clinical and experimental studies, and relevant reviews were considered. Key study designs and results were checked against primary reports. Study selection was not systematic, no formal risk-of-bias assessment was undertaken, and the review may omit relevant studies.

We use descriptive evidence categories rather than recommendation classes or letter grades. “Guideline-supported” identifies a specific practice supported by a cited guideline or consensus document; “randomized evidence” refers to a tested intervention and its measured endpoint; “prospective observational evidence” and “retrospective evidence” describe non-randomized human studies; and “mechanistic/extrapolative evidence” identifies experimental findings or reasoning transferred between settings. These categories describe the source of support, not a hierarchy of recommendation strength. Different categories may apply to different claims within one treatment group. “Authors’ proposed framework” separately identifies surveillance schedules, imaging thresholds, or decision aids without prospective validation for that use. Evidence for a drug or a CMR endpoint does not by itself validate a CMR-guided treatment strategy. During revision, an AI-based tool assisted with wording, consistency checks, and drafting revisions; details are disclosed in the Acknowledgments.

## 2. Pathophysiology of CTRCD and the Rationale for Early Detection

### 2.1. From a Static Dichotomy to a Dynamic Framework

The distinction between irreversible “type I” anthracycline injury and reversible “type II” trastuzumab injury is incomplete because mechanisms and clinical recovery overlap [13,14]. The European Society of Cardiology (ESC) and International Cardio-Oncology Society (IC-OS) instead classify CTRCD by symptoms, LVEF, echocardiographic GLS, and cardiac biomarkers [15,16]. These definitions recognize asymptomatic dysfunction before overt heart failure. CMR mapping may add information about tissue abnormalities, but mapping findings are not part of the formal severity definitions. Management also depends on the anticancer agent; the same LVEF does not imply the same treatment decision across therapies.

### 2.2. The Temporal Cascade of Myocardial Injury

Anthracycline injury provides a useful, but incomplete, model of evolving myocardial damage. Doxorubicin-related oxidative stress, topoisomerase IIβ-mediated DNA damage, and mitochondrial dysfunction contribute to cardiomyocyte injury [17,18]. In a serial CMR study in pigs, isolated T2 prolongation at week 6 corresponded to intracellular edema on histology; stopping doxorubicin at that stage prevented subsequent fibrosis and ventricular dysfunction [19]. Human cohorts have also shown early changes in mapping parameters, although these are often small and transient [20]. The sequence from edema to extracellular expansion and functional impairment should therefore be viewed as a conceptual model rather than a fixed biological order. Troponin release does not establish reversibility, and individual CMR parameters cannot identify a histological stage with certainty. An assessment around human chemotherapy cycles 2–3 is a proposed translational hypothesis, not an established equivalent of the animal week-6 window. Figure 1 summarizes these distinctions.

### 2.3. Integrating Biomarkers and Imaging

Cardiac CARE did not demonstrate an LVEF benefit from its troponin-guided treatment strategy, but it did not test whether tissue-guided treatment is superior [6,7]. In a prospective study of 136 women receiving sequential anthracycline and trastuzumab therapy, T1, T2, and ECV changes were generally modest and transient; 27% developed CTRCD by functional criteria, while no new LGE was observed [20]. These findings illustrate the imperfect correspondence among biomarkers, tissue measurements, and functional outcomes. A small isolated change should be confirmed and interpreted in context rather than assumed to represent either harmless permeability change or irreversible injury.

### 2.4. Treatment-Specific Mechanisms of Injury

The seven treatment groups differ in their predominant mechanisms: anthracycline-related oxidative and DNA injury; disruption of cardiomyocyte HER2 signaling; VEGF-inhibitor-associated hypertension and vascular dysfunction; ICI-related immune myocardial injury; radiation-associated vascular, myocardial, valvular, and pericardial disease; CAR-T-associated cytokine release syndrome (CRS); and proteasome-inhibitor-associated cardiovascular stress [13,15,21,22]. These mechanisms can overlap with pre-existing disease and concomitant treatment. Antibody–drug conjugates and bispecific antibodies warrant separate study. Their payloads, targets, and systemic toxicities mean that findings from trastuzumab or CAR-T cohorts cannot be transferred directly. Dedicated CMR data remain limited; any predicted tissue pattern should be identified as mechanistic extrapolation rather than an established signature.

### 2.5. Clinical Consequences of Diagnostic Error

The consequences of a false-positive or false-negative result depend on the treatment and clinical presentation. An unnecessary interruption may reduce the delivery of effective therapy [3], whereas missed ICI myocarditis can progress rapidly [23,24]. Neither biomarker release nor a mapping abnormality is sufficient in isolation to resolve this balance. Interpretation should integrate symptoms, ECG findings, biomarkers, ventricular function, treatment exposure, and the reliability of serial measurements. This approach also avoids assuming that all early abnormalities are reversible or that all persistent abnormalities require treatment withdrawal.

## 3. Multiparametric CMR Measurements

Multiparametric CMR describes ventricular function, tissue water, extracellular expansion, focal enhancement, and perfusion. These measurements are complementary and are not interchangeable with histology. Table 1 summarizes interpretation requirements; Figure 2 links the principal uses of each technique to descriptive evidence categories.

### 3.1. T2 Mapping and T2-Weighted Imaging

T2 mapping is sensitive to increased myocardial water, including intracellular and interstitial edema [9,19]. T2-weighted short-tau inversion recovery (T2-STIR) imaging can provide an alternative when mapping is unavailable. T2 elevation has different implications in an asymptomatic patient receiving anthracyclines and in a patient with suspected ICI myocarditis [20,23]. An isolated change does not establish reversibility or mandate treatment interruption. Conversely, a normal T2 does not exclude inflammation, particularly when imaging is early or sensitivity is limited. Clinical context and the other CMR findings remain essential.

### 3.2. Extracellular Expansion Assessed with Native T1 and ECV

Native T1 can increase with edema, fibrosis, or infiltration. ECV estimates the extracellular distribution volume of gadolinium and is associated with diffuse interstitial expansion, including fibrosis, but is not collagen-specific [9,28]:

$$\mathrm{ECV}\;=\;\left(1-\mathrm{Hct}\right)\;\times \frac{\Delta R1_{\mathrm{myocardium}}}{\Delta R1_{\mathrm{blood}}}$$

Here, ΔR1 is the change in the longitudinal relaxation rate (1/T1) from before to after contrast administration in myocardium or blood. Hct is expressed as a fraction; the calculated ECV fraction is multiplied by 100 when reported as a percentage. Two issues are particularly important when interpreting serial ECV in patients receiving cancer therapy.

First, an absolute ECV increase of at least 3 percentage points is a candidate risk signal, not a diagnostic cutoff for irreversible fibrosis. In the STOP-CA substudy, 127 participants had analyzable paired contrast scans. The proportion with an ECV rise of at least 3 percentage points was 8% with atorvastatin and 29% with placebo (odds ratio 0.20; 95% CI, 0.06–0.59); this rise was associated with greater LVEF decline [27]. A separate non-oncologic cohort linked higher ECV to adverse outcomes, supporting prognostic relevance without validating a serial oncology treatment threshold [29]. Serial ECV should be assessed against local test–retest reproducibility and the minimal detectable change (MDC). T2, native T1, hematocrit, and clinical findings help assess alternative explanations, but normal T2 cannot exclude residual or non-collagen extracellular expansion. A reproducible rise may be interpreted as a higher-risk signal of extracellular expansion/remodeling; CMR cannot provide definitive histopathological attribution in this setting. Neither a 3-percentage-point rise nor persistence after T2 normalization is a stand-alone indication to interrupt treatment.

Second, ECV depends on hematocrit (Hct). Anemia, fluid shifts, and differences in blood-sampling conditions can affect interpretation, while synthetic-Hct models may introduce clinically relevant error [34,35,36]. We favor measured Hct obtained close to the scan, preferably on the same day, with consistent timing and posture across visits. The sampling protocol should account for changes during supine rest; a cited methodological study favored sampling while the patient remained supine after the examination [36]. The evidence does not support a universal requirement for a seated sample within 30 min before scanning. Synthetic Hct should not be substituted without local validation, particularly during treatment-related anemia. Reports should document sampling conditions and avoid assigning biological meaning to changes that are within measurement uncertainty.

### 3.3. Myocardial Deformation Assessed by CMR Feature Tracking

CMR feature tracking (CMR-FT) estimates myocardial strain from cine images without additional acquisitions and may be useful when echocardiographic image quality is limited [30,31]. The greater than 15% relative decline in GLS used in ESC/IC-OS CTRCD definitions derives from echocardiographic speckle tracking [15,16]. It is not a prospectively validated CMR-FT treatment threshold. Applying it to CMR-FT is an extrapolation because tracking methods, temporal resolution, and software algorithms differ. Serial CMR-FT should use the same analysis platform and version, consistent contouring, and local reproducibility estimates; a numerical change should exceed the local MDC before it is interpreted as progression. Training and measurement consistency are relevant for both modalities [37]. A relative decline refers to reduced strain magnitude, rather than the sign of the negative GLS value.

### 3.4. Late Gadolinium Enhancement and Its Limitations in ICI Myocarditis

LGE identifies regions with expanded contrast distribution compatible with myocardial injury or scar, but it does not independently establish irreversible necrosis. In a registry of 103 patients with ICI myocarditis, LGE was present in 48%; detection was less frequent when CMR was performed within 4 days of admission [23]. This association with timing does not establish the mechanism of a negative scan. In a separate mapping analysis, abnormal T1 and T2 were reported in 78% and 43%, respectively, of patients with the relevant measurements [26]. The 2018 modified Lake Louise criteria combine at least one T2-based marker of edema with at least one T1-based marker of injury, such as elevated native T1 or ECV, or non-ischemic LGE, to provide stronger CMR evidence of myocardial inflammation [25]. One positive marker may still support inflammation in an appropriate clinical setting. These are imaging criteria, not a self-contained definition of definite ICI myocarditis; a negative or incomplete CMR examination does not exclude the clinical diagnosis.

### 3.5. First-Pass Perfusion and MPRI

Stress perfusion and the myocardial perfusion reserve index (MPRI) can assess impaired perfusion reserve. They may help investigate ischemic or microvascular disease after VEGF-pathway inhibition or chest radiotherapy [22]. However, reduced reserve is not specific for treatment-related microvascular injury, and coronary artery disease, loading conditions, and technical factors must be considered. Prospective data validating MPRI-guided treatment decisions in these settings are limited.

### 3.6. Standardization Before Interpretation

Mapping values vary with field strength, sequence, vendor, and analysis method [9,38]. Local reference ranges and quality control are therefore necessary. A cohort of 30–50 healthy volunteers may be a pragmatic starting point, but it is not a universal minimum and may be insufficient for age- or sex-specific ranges. For an approximately normal reference distribution, an elevated value can be expressed as:

$$z=\frac{x-\mu }{\sigma },\;z\;>2$$

Here, x is the measured value, μ the local reference mean, and σ the reference standard deviation. A z-score above 2 is a practical flag for an elevated value, not a disease-specific diagnostic cutoff. Serial change should also exceed measurement uncertainty, estimated from local test–retest data for that parameter [39,40]:

$$\mathrm{MDC}\;=\;1.96\;\times \;\sqrt{2}\;\times \;\mathrm{SEM},\;\mathrm{SEM}\;=\;\mathrm{SD}\;\times \;\sqrt{1-\mathrm{ICC}}$$

Here, SEM is the standard error of measurement, SD the relevant measurement standard deviation, and ICC the intraclass correlation coefficient. The MDC formula assumes appropriate repeatability data; its value should not be borrowed uncritically from another platform or population. Serial scans should use the same scanner, field strength, protocol, and software version where possible. If systems change, comparability must be established before absolute differences are attributed to biology. Diffusion tensor imaging and four-dimensional flow remain investigational for this application.

## 4. CMR Findings Across Seven Anticancer Treatment Groups

The following sections distinguish observed findings from anticipated mechanisms and proposed clinical uses. Table 2 pairs treatment-specific observations with their evidence sources and the status of suggested CMR schedules. Table 3 separates guideline-supported management principles from proposed imaging aids. Figure 3 provides a qualitative overview without numerical scores or recommendation grades.

### 4.1. Anthracyclines

Doxorubicin, daunorubicin, and epirubicin are used in breast cancer, lymphoma, leukemia, and sarcoma. Oxidative stress and topoisomerase IIβ-mediated injury contribute to cumulative myocardial damage [17,18]. Experimental studies support an early edema phase, while human studies report changes in mapping, myocardial mass, strain, and function [19,28,64]. The timing and reversibility vary. A CMR examination around cycles 2–3 is a proposed research-oriented checkpoint rather than a validated clinical window, and cumulative dose does not map uniformly to cycle number. The ECV association in STOP-CA supports further evaluation of extracellular expansion as a risk marker [27]; it does not identify with certainty which individual patients will progress.

Randomized prevention studies have produced differing results: PRADA found a modest early LVEF benefit with candesartan, whereas CECCY and Cardiac CARE did not meet their primary cardioprotection objectives [6,41,42]. In STOP-CA, atorvastatin reduced the trial-defined LVEF endpoint from 22% to 9% in lymphoma patients [32]. These findings concern specific interventions and populations, not the efficacy of a CMR-guided treatment pathway. The published RESILIENCE design describes a multicenter study of remote ischemic conditioning and CMR methods; a design paper cannot establish treatment efficacy or implementation success [69]. LGE is not consistently present after anthracyclines, and other cardiac causes should be considered when it is found [65]. Ventricular volumes, mass, right-ventricular function, and diffuse tissue measurements may provide additional context [64,66]. Decisions about interruption should follow clinical severity and treatment-specific guidance, with mapping changes contributing to reassessment rather than acting as independent stopping rules.

### 4.2. Anti-HER2 Antibodies

Trastuzumab and pertuzumab can impair myocardial function through disruption of HER2-related signaling. Recovery is possible, but persistent dysfunction occurs, especially with prior anthracycline exposure or other cardiac disease [14,21]. CMR may show functional or deformation changes without new LGE; a purely reversible phenotype should not be assumed [20]. In SAFE-HEaRt, 27 of 30 evaluable patients with asymptomatic LVEF 40–49% completed planned HER2-targeted therapy without the study-defined cardiac endpoint while receiving cardioprotective medication and close follow-up [43]. Long-term follow-up remained reassuring in the small selected cohort [44]. SAFE-HEaRt used echocardiography at baseline, 6 and 12 weeks, and then every 12 weeks; it did not validate CMR every 3–4 weeks. SCHOLAR provides additional prospective, single-arm evidence for carefully monitored continuation [45]. Neither study establishes absolute exclusions based on a prior doxorubicin dose of 300 mg/m^2^ or an isolated CMR volume change. Prevention studies also differ: Guglin and colleagues evaluated lisinopril and carvedilol, MANTICORE evaluated perindopril and bisoprolol, and a separate trial evaluated candesartan [46,47,48]. These results support individualized cardio-oncology care rather than uniform prophylaxis or fixed CMR schedules.

### 4.3. VEGF-Pathway Inhibitors

Sunitinib, sorafenib, and related tyrosine kinase inhibitors are used in renal cell carcinoma, hepatocellular carcinoma, and gastrointestinal stromal tumors. Hypertension, vascular effects, and ventricular dysfunction contribute to their cardiovascular toxicity [22,49]. CMR can clarify ventricular function and assess selected questions about ischemia or tissue injury. Concentric remodeling or reduced perfusion reserve is not a validated drug-specific signature and cannot reliably separate direct toxicity from hypertension on its own. Blood-pressure control and evaluation of ischemia or heart failure take priority. Interruption and resumption depend on the severity of toxicity, response to treatment, and oncologic benefit [15].

### 4.4. Immune Checkpoint Inhibitors

ICIs targeting programmed cell death protein 1 (PD-1), its ligand PD-L1, or cytotoxic T-lymphocyte-associated protein 4 (CTLA-4) can cause myocarditis across multiple cancer types. Severe cases may progress rapidly, and early registries and pharmacovigilance reports described substantial mortality [24,50,67,68]. These selected data should not be treated as a uniform contemporary case-fatality estimate. Presentation is often early; one registry reported a median onset of 34 days, although later disease occurs [24]. CMR may demonstrate edema or non-ischemic injury, but LGE and mapping can be negative. The reported association between native T1 and major adverse cardiovascular events was derived from a selected registry and requires external validation [23,26].

Routine serial CMR screening is not established for asymptomatic ICI recipients. New cardiac symptoms, arrhythmia, conduction disease, or a troponin rise should prompt urgent evaluation and interruption of ICI therapy while suspected myocarditis is investigated [15]. When myocarditis is considered likely, high-dose intravenous methylprednisolone (500–1000 mg daily for 3–5 days) is recommended; treatment should not wait for CMR completion, particularly in unstable patients [15]. Steroid-refractory disease requires specialist-directed escalation; the evidence for agents such as abatacept remains limited [61]. CMR findings should be integrated with the clinical presentation, ECG, biomarkers, ventricular function, and associated immune-related adverse events; endomyocardial biopsy may be needed when uncertainty persists [15,62]. Earlier corticosteroid treatment has been associated with better outcomes in retrospective data [63]. Myositis or myasthenic features also require prompt evaluation. Decisions about permanent discontinuation or exceptional rechallenge should be made by a cardio-oncology multidisciplinary team (MDT).

### 4.5. Chest Radiotherapy

Chest radiotherapy can affect coronary arteries, microvasculature, myocardium, valves, and pericardium [22,51,52,53,54]. CMR can characterize ventricular function, scar, perfusion, and pericardial disease in selected patients. Findings should be interpreted alongside the radiation field and cardiac dose distribution, but field-related abnormalities do not exclude coronary artery disease. Some changes appear early, whereas clinically apparent disease may emerge years or decades later. The association between mean cardiac dose and subsequent ischemic events supports risk-based long-term follow-up [52,54]. CMR complements, rather than replaces, guideline-based surveillance and investigations directed by symptoms. A fixed CMR schedule at years 1, 3, 5, and 10 has not been validated.

### 4.6. CAR-T Cell Therapy

Cardiovascular complications after CAR-T therapy often occur during CRS and may include hypotension, arrhythmia, and ventricular dysfunction [55,56]. The supporting cohorts are mainly retrospective and do not establish a characteristic diffuse subendocardial CMR edema pattern. Echocardiography and hemodynamic assessment are the practical first-line tests during instability. CMR may be useful after stabilization when the mechanism remains uncertain, but transport or imaging must not delay CRS treatment and supportive care. Tocilizumab and corticosteroids are used according to CRS severity and the treatment protocol [15].

### 4.7. Proteasome Inhibitors

Proteasome inhibitors, particularly carfilzomib, are associated with hypertension, heart failure, and other cardiovascular events in multiple myeloma [57,58]. CMR may clarify unexplained dysfunction or investigate coexisting cardiac amyloidosis. Elevated native T1 or ECV and characteristic LGE can support suspicion of amyloid infiltration, but CMR alone cannot distinguish AL amyloidosis from all other amyloid types or establish proteasome-inhibitor causality [59]. Laboratory and, when indicated, tissue evaluation remain necessary. Prospective biomarker data support careful risk assessment and monitoring [58]; they do not establish routine baseline CMR for every high-risk patient as a standard.

Across these groups, the strongest randomized evidence concerns selected cardioprotective interventions with functional endpoints. Much of the tissue-characterization evidence is observational, and some proposed signatures rely on mechanistic extrapolation. The anthracycline injury sequence cannot be applied uniformly to other therapies. CMR is most likely to be useful when it answers a specific question about ventricular function, myocardial inflammation, ischemia, or competing diagnoses that remains unresolved after first-line assessment.

## 5. Risk-Adapted CMR Surveillance

CMR complements echocardiography and biomarkers when first-line findings are inconclusive, measurement quality is inadequate, or tissue characterization may change management. The proposed pathway allocates CMR according to baseline risk and the clinical question, while allowing urgent presentations to bypass routine surveillance (Figure 4). Table 4 summarizes this approach. Its effect on cardiovascular events, cancer treatment completion, and cost-effectiveness has not been tested.

### 5.1. Baseline Risk Stratification

Baseline assessment should follow the treatment-specific HFA/IC-OS framework and guideline recommendations for history, examination, ECG, echocardiography, and biomarkers [15,60,70]. Cumulative anthracycline exposure, cardiac radiation dose, pre-existing cardiovascular disease, baseline function, and other risk factors inform surveillance intensity. Treatment categories should not be assigned the same risk solely because they involve dual HER2 blockade or combined immunotherapy. In selected high-risk patients, baseline CMR may be considered if echocardiography is inadequate or tissue characterization addresses an unresolved question. Routine CMR solely on the basis of a risk category remains a proposed extension.

### 5.2. Low-Risk Patients

For patients at low baseline risk, surveillance generally remains based on clinical assessment, echocardiography, and biomarkers where indicated. CMR is reserved for unresolved diagnostic questions or inadequate imaging. Cardiac CARE cautions against assuming benefit from a particular biomarker-triggered drug strategy [6]; it does not directly compare selective with routine CMR surveillance. Avoiding unnecessary testing is reasonable, but the resource benefits of this proposed pathway require evaluation.

### 5.3. Medium-Risk Patients

Patients at medium risk on a treatment-specific HFA/IC-OS assessment require the surveillance frequency recommended for that therapy [15,70]. A universal schedule at each or every second cycle is inappropriate across all seven treatment groups. CMR may clarify an unexplained functional change, a poor acoustic window, or clinically meaningful disagreement between imaging and biomarkers. The choice and timing should depend on whether additional information is likely to alter care, rather than on an unvalidated numerical trigger.

### 5.4. Proposed Checkpoints for Selected High-Risk Patients

For selected high- or very-high-risk patients, we propose evaluating CMR at baseline, during treatment, or at treatment completion when an unresolved question warrants tissue characterization or more reproducible functional assessment. A mid-treatment anthracycline scan around cycles 2–3 remains a hypothesis for prospective testing, not a required checkpoint [19]. Cumulative-dose thresholds used in guideline surveillance do not automatically mandate CMR [15]. A consistent core dataset can include cine imaging, T2 and native T1 mapping, CMR-FT, and ECV when contrast is appropriate. Reports should provide raw values, local reference ranges, image quality, and the uncertainty of serial change.

### 5.5. Reassessment When Findings Are Uncertain

For an asymptomatic, clinically stable patient without concern for an acute syndrome, an unexpected biomarker or imaging result can be checked for technical error, baseline context, and alternative causes before escalation. Repeat testing should occur at an interval appropriate to the treatment and the magnitude and trajectory of change. We do not propose a universal troponin range or a 72-h-to-one-week waiting rule. In particular, a new troponin rise during ICI treatment, ischemic symptoms, arrhythmia, conduction disturbance, or hemodynamic instability requires prompt assessment rather than entry into a routine repeat-testing pathway [15].

When echocardiographic windows are limited, contrast-enhanced or three-dimensional echocardiography may improve assessment; CMR is appropriate if uncertainty persists and it can be performed safely. Clinically important discordance means that the available results support different management choices, not merely that two measurements differ. For suspected myocarditis, urgent CMR may assist diagnosis once feasible, but a fixed 48-h imaging target is not a condition for treatment. Access limitations should be addressed through clinical triage and alternative tests, without delaying necessary cardiac care or automatically postponing anticancer treatment.

### 5.6. Evidence for Intervention After Subclinical Changes

SUCCOUR-MRI followed 333 at-risk patients receiving anthracyclines; 105 who developed an isolated greater than 12% relative decline in echocardiographic GLS were randomized to neurohormonal cardioprotection or usual care [33]. The 12-month CMR-LVEF decline was smaller with cardioprotection (−2.5 versus −5.6 percentage points; *p* = 0.009). LVEF-defined CTRCD occurred in 1 of 49 versus 6 of 56 patients (*p* = 0.075), a difference that was not statistically significant. The trial supports intervention after a specific echocardiographic strain signal in a selected population. It does not validate a CMR-FT GLS cutoff, mapping-guided treatment, or interruption of anticancer therapy. The original SUCCOUR trial and its follow-up also show that conclusions depend on the endpoint and follow-up period [71,72].

Cardiac CARE and SUCCOUR-MRI used different selection criteria, triggers, and designs [6,33]. Their differing findings cannot be attributed with certainty to reversible troponin leakage versus structural injury. Together, they support further study of how patient risk and the choice of trigger affect treatment benefit. Extrapolation from an echocardiographic strain-guided intervention to a CMR tissue-guided strategy remains untested.

### 5.7. Survivorship and Special Populations

Long-term follow-up should be based on treatment exposure, baseline and end-of-treatment risk, symptoms, and residual abnormalities [15,53,54]. Echocardiography remains central; CMR can answer selected questions about scar, perfusion, ventricular function, or inconclusive findings. Evidence does not establish a routine CMR schedule at years 1, 3, 5, and 10. Childhood and adolescent survivors require dedicated guidance rather than direct application of adult pathways [73,74]. In advanced renal impairment, a contrast-free examination using cine, native T1/T2, and CMR-FT may be useful. Any decision to administer gadolinium should consider the specific agent, renal function, diagnostic benefit, and local safety policy rather than an absolute prohibition based on an estimated glomerular filtration rate alone.

### 5.8. Health Economics and Access

Shorter acquisitions, free-breathing cine, and automated analysis may improve feasibility, particularly for frail patients. The RESILIENCE design includes evaluation of CMR methods but does not establish the clinical effectiveness or cost of a complete abbreviated surveillance pathway [69]. No formal economic evaluation supports the pathway proposed here. Such work should measure diagnostic yield, access, downstream testing, treatment delays, and both cardiac and oncologic outcomes. Claims that a CMR scan is necessarily cheaper than a treatment delay or admission are not supported by the available evidence.

## 6. Integrating CMR into Clinical Decisions

CMR findings should be interpreted within treatment-specific management rather than linked automatically to a prescription. Figure 5 separates assessment of formal CTRCD severity, additional tissue information, and decisions about cardioprotection or anticancer treatment. The proposed imaging criteria below are decision aids for MDT review; they are not prospectively validated mandatory criteria.

### 6.1. Cardioprotection and Treatment Modification

The first decision is whether established CTRCD or the overall cardiovascular risk warrants cardioprotective treatment. For mild asymptomatic CTRCD, an ACE inhibitor or ARB and/or a beta-blocker may be considered according to the anticancer regimen and clinical guidance, with continued therapy and closer surveillance where appropriate [15]. A small T1 or T2 change within measurement uncertainty is not, by itself, an indication for GDMT. For example, an asymptomatic patient receiving anthracyclines with LVEF 56% and a reproducible 16% relative decline in echocardiographic GLS meets the strain component of mild asymptomatic CTRCD; the decision does not require abnormal mapping. This example must not be interpreted as validation of an identical CMR-FT threshold.

The second decision is whether anticancer therapy should be interrupted, modified, or continued. Symptoms, functional severity, arrhythmia, suspected myocarditis, cumulative exposure, and expected oncologic benefit carry greater decision weight than an isolated mapping value [15]. New LGE, T2 elevation, or an ECV increase may warrant further investigation, but none has a universally validated stand-alone stopping threshold. An ECV rise of 4 percentage points that exceeds the local MDC should prompt review of acquisition quality, Hct, other CMR findings, and the clinical course [27]. Normal T2 does not convert that result into proof of fibrosis. Urgent clinical syndromes should be treated without waiting for structural confirmation on CMR.

### 6.2. Treatment-Specific Continuation Under Close Surveillance

Continuation despite cardiac dysfunction has the clearest prospective support in selected patients receiving HER2-targeted therapy. SAFE-HEaRt and SCHOLAR support feasibility under specialist supervision, cardioprotective treatment, and close monitoring, but their small single-arm designs limit generalization [43,44,45]. ESC guidance allows continued HER2 therapy in asymptomatic moderate CTRCD with appropriate treatment and surveillance [15]. Prior anthracycline exposure, ischemic heart disease, or progressive ventricular enlargement should inform risk assessment; none provides a universally validated numerical exclusion criterion. These observations must not be generalized to fulminant ICI myocarditis or unstable CRS-associated cardiac dysfunction. Severe or rapidly evolving presentations require urgent management, while subsequent treatment decisions remain specific to the agent and clinical course.

### 6.3. ESC/IC-OS Definitions and the Separate Role of Mapping

The ESC/IC-OS asymptomatic CTRCD definitions use the following criteria [15,16]. Mild: LVEF ≥ 50% with a new relative decline in echocardiographic GLS > 15% and/or a new rise in cardiac biomarkers. Moderate: a new LVEF reduction of ≥10 percentage points to 40–49%, or a reduction of <10 percentage points to 40–49% accompanied by a new relative GLS decline > 15% and/or a new biomarker rise. Severe: a new LVEF reduction to <40%. Biomarker changes require assay-appropriate thresholds and clinical interpretation. Mapping abnormalities are not included in these definitions and should be reported separately. Symptomatic CTRCD is classified by the clinical heart-failure syndrome and the need for treatment escalation, not by LVEF alone. Myocarditis is a separate diagnosis and should not be equated with severe CTRCD merely because the modified Lake Louise criteria are met. Management then follows treatment-specific guidance: anthracycline-associated moderate or severe asymptomatic CTRCD generally requires interruption, whereas selected asymptomatic patients with LVEF 40–49% may continue HER2-targeted therapy under specialist care [15].

### 6.4. Reassessment After Deterioration or Incomplete Recovery

New symptoms or further LVEF decline during continued therapy should trigger prompt reassessment. Interruption is recommended for severe asymptomatic HER2-related CTRCD and for clinically significant symptomatic dysfunction according to guideline-defined severity [15]. An isolated mild symptom should not automatically be relabeled as severe CTRCD requiring permanent withdrawal. If recovery remains incomplete after heart-failure treatment, the MDT should reconsider the anticancer options, reversible contributors, and the patient’s preferences. Persistent LGE alone is not a validated indication for permanent discontinuation, and failure to meet an arbitrary 8–12-week imaging target should not predetermine that decision.

### 6.5. Proposed CMR-Informed Rechallenge Considerations

Our proposed CMR-informed framework considers clinical stability, recovery or stabilization of ventricular function, improvement in serial strain, assessment of suspected active inflammation, and documented MDT agreement. These considerations have not been prospectively validated as mandatory restart criteria. LVEF ≥ 50%, CMR-FT GLS within 10% of baseline, and T2 within the local reference range may be recorded as exploratory recovery targets, but all three need not be achieved before every restart, and normal T2 does not prove complete inflammatory resolution. A CMR reassessment at 4–6 weeks after restarting may be considered when it addresses a specific question; it is not a mandatory schedule. Treatment-specific guidance takes precedence. For HER2-targeted therapy, resumption can be considered after symptom resolution and LVEF recovery to ≥40%, ideally ≥50%, with heart-failure treatment and close echocardiographic and biomarker surveillance [15]. Rechallenge after ICI myocarditis requires a separate, individualized MDT assessment of recovery, prior severity, cancer benefit, alternatives, and patient preferences; the proposed numerical CMR targets do not establish its safety.

In advanced cancer with limited effective alternatives, the balance between cardiovascular risk and cancer control may favor a carefully monitored treatment strategy despite incomplete cardiac recovery [15]. Such decisions require a shared assessment of individual circumstances; a fixed LVEF range does not establish safety. The MDT should document the treatment options, anticipated benefit, cardiac risk, monitoring plan, and the patient’s informed preferences. Deterioration requires renewed assessment and appropriate interruption; permanent discontinuation should not follow automatically from a single numerical threshold across all therapies.

### 6.6. Cardioprotective Treatment

Treatment of established heart failure or clinically significant CTRCD should follow contemporary guideline-directed therapy as tolerated: an ACE inhibitor, ARB, or angiotensin receptor–neprilysin inhibitor; a beta-blocker; a mineralocorticoid receptor antagonist; and a sodium-glucose cotransporter-2 (SGLT2) inhibitor when indicated [15]. The established role of SGLT2 inhibitors in heart failure should be distinguished from their investigational use specifically to prevent anthracycline cardiotoxicity. Observational oncology studies are encouraging but cannot establish prevention efficacy [75,76]. In STOP-CA, atorvastatin reduced an LVEF-defined endpoint, whereas the difference in heart-failure events (3% versus 6%) was not statistically significant [32]. The ECV substudy provides mechanistic and prognostic information, not a tissue-guided prescription rule [27]. Mixed findings from other prevention trials support attention to population, endpoint, and duration of follow-up [6,41,42,46,47,48,77,78]. When anthracyclines are reconsidered after toxicity, dose adjustment, liposomal preparations, and dexrazoxane are options for MDT discussion in accordance with clinical guidance [15,79].

## 7. Limitations and Future Directions

Table 5 summarizes selected studies and their limits of inference. Several limitations constrain clinical application. This is an English-language narrative review without systematic selection or formal risk-of-bias assessment. Tissue-characterization studies are often small, selected, and heterogeneous in treatment exposure and imaging methods. Randomized trials with CMR endpoints have mainly assessed cardiac surrogates; they have not established that the proposed CMR-guided pathway improves treatment completion, RDI, progression-free survival (PFS), or overall survival (OS) [32,33]. Evidence categories describe study sources rather than formally graded certainty. The 3-percentage-point ECV signal lacks serial histological validation and a validated intervention threshold, and normal T2 does not resolve its histological specificity [27,29]. CMR-FT thresholds and the proposed rechallenge targets require prospective validation. Scanner and software differences, local reference data, Hct sampling, and measurement uncertainty affect serial interpretation [9,34,35,36,39]. Access, scan duration, arrhythmia, frailty, renal impairment, and device-related safety or artifacts also limit feasibility. These constraints support selective use of CMR to answer defined clinical questions.

Future studies should evaluate standardized acquisition and analysis across centers, with blinded outcome assessment and explicit reporting of measurement reproducibility. Contrast-free protocols and shorter examinations need validation in the patients most likely to require them. Prospective cohorts should include older adults, children, diverse populations, and recipients of antibody–drug conjugates, bispecific antibodies, and newer cellular therapies. Automated analysis and combined imaging–biomarker models should be tested externally before they inform treatment decisions. Trials should compare a prespecified CMR-informed strategy with guideline-based care and measure cardiac events, treatment completion, RDI, PFS, OS, patient burden, and cost. The RESILIENCE design illustrates one approach to incorporating CMR into multicenter research, but does not itself validate the pathway proposed here [69].

## 8. Conclusions

CMR provides complementary assessment of ventricular function and myocardial tissue when first-line surveillance leaves a clinically important question unresolved. Mapping and LGE can help characterize injury, and CMR-FT offers additional functional information, but no single measurement establishes reversibility or the need to stop cancer treatment. A negative CMR does not exclude ICI myocarditis. The echocardiographic GLS threshold used in formal CTRCD definitions should remain distinct from unvalidated CMR-FT thresholds.

The surveillance schedules and CMR-informed rechallenge considerations described here are proposed decision aids. A reproducible ECV increase is a risk signal, not proof of collagen deposition or an independent treatment threshold. Clinical severity, treatment-specific guidance, oncologic benefit, and patient preferences remain central to MDT decisions. Prospective evaluation is needed before this framework can be recommended as a strategy for improving cardiovascular or cancer outcomes.

## Figures and Tables

**Figure 1:**
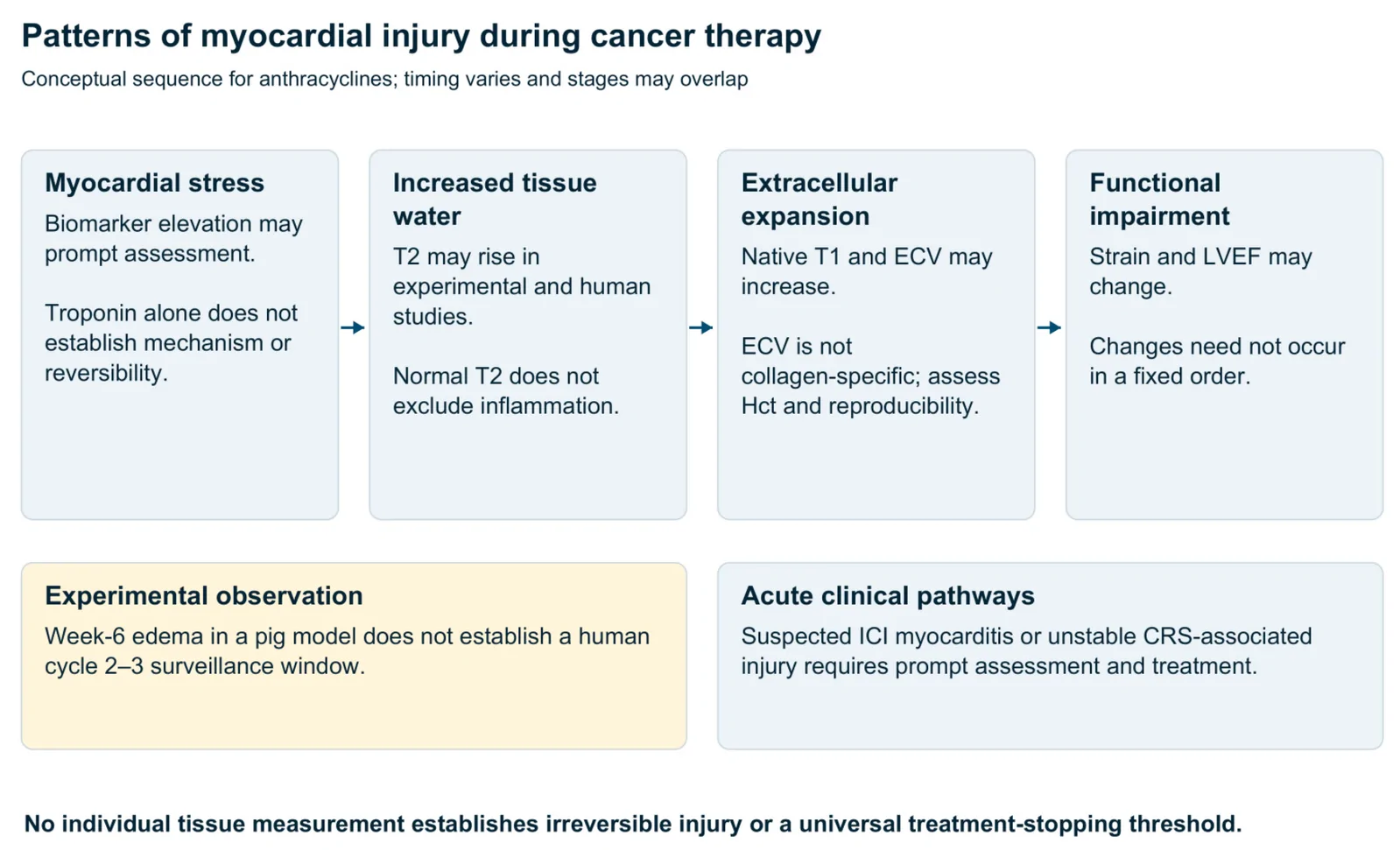
Conceptual patterns of cancer therapy-related myocardial injury. The anthracycline sequence is informed by experimental and human studies [19,20]; stages may overlap, and their timing is not uniform. The animal week-6 observation does not establish a human cycle-specific surveillance window. Acute ICI myocarditis and CAR-T-associated injury require separate clinical pathways. ECV expansion is not specific for fibrosis, and troponin elevation does not establish reversibility. CMR, cardiovascular magnetic resonance; ECV, extracellular volume fraction; LVEF, left ventricular ejection fraction; ICI, immune checkpoint inhibitor; CAR-T, chimeric antigen receptor T-cell therapy.

**Figure 2:**
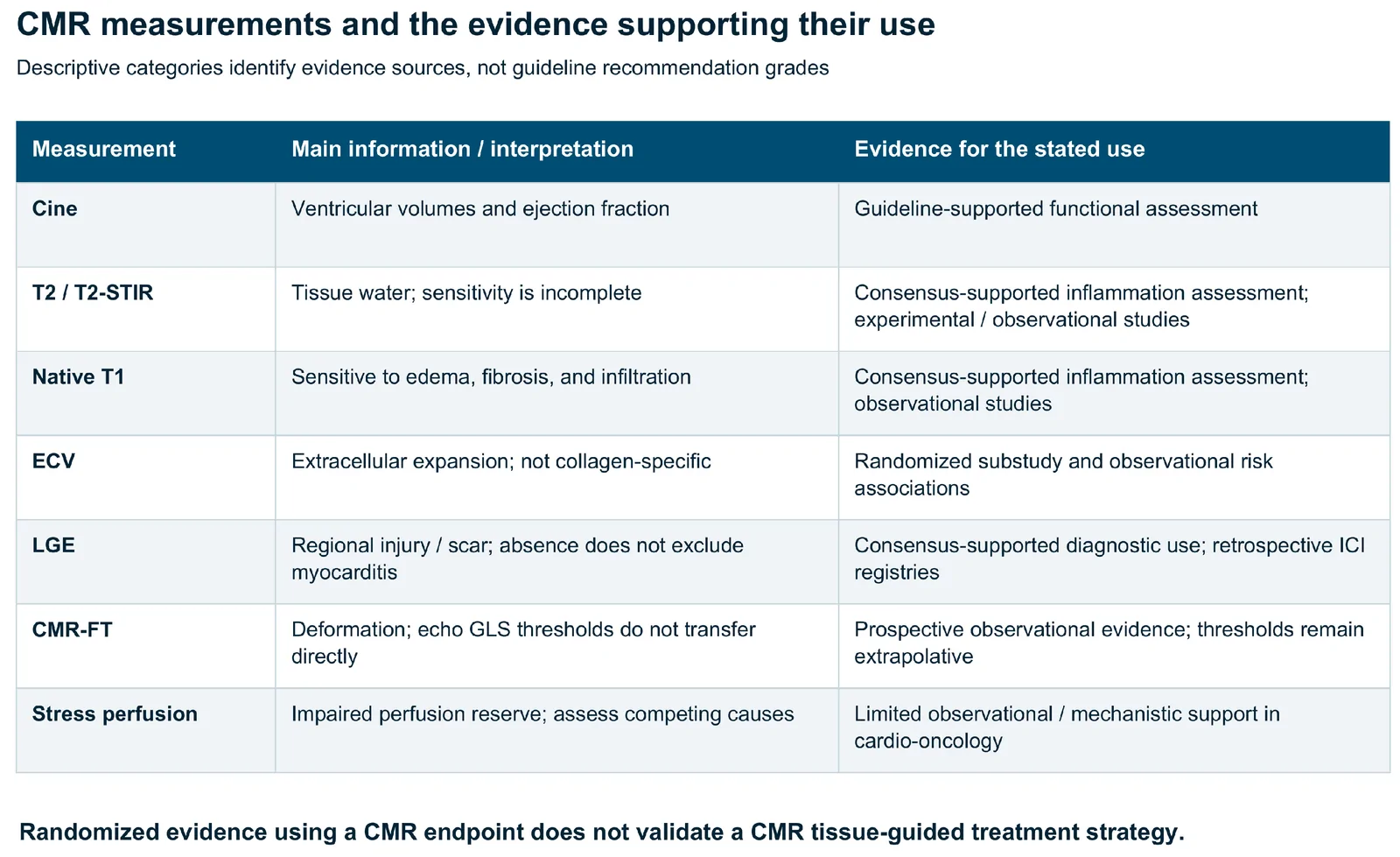
Principal CMR measurements and their supporting evidence. Descriptive categories identify the source of support for the stated use; they are not recommendation grades or estimates of diagnostic accuracy. Guideline-supported use in the evaluation of suspected myocarditis does not establish that routine mapping surveillance improves outcomes. Randomized trials using CMR endpoints have generally tested cardioprotective drugs or echocardiographic strain-guided intervention, not a CMR tissue-guided treatment strategy [8,9,15,19,20,23,25,27,30,31,32,33]. CMR-FT, CMR feature tracking; GLS, global longitudinal strain; LGE, late gadolinium enhancement.

**Figure 3:**
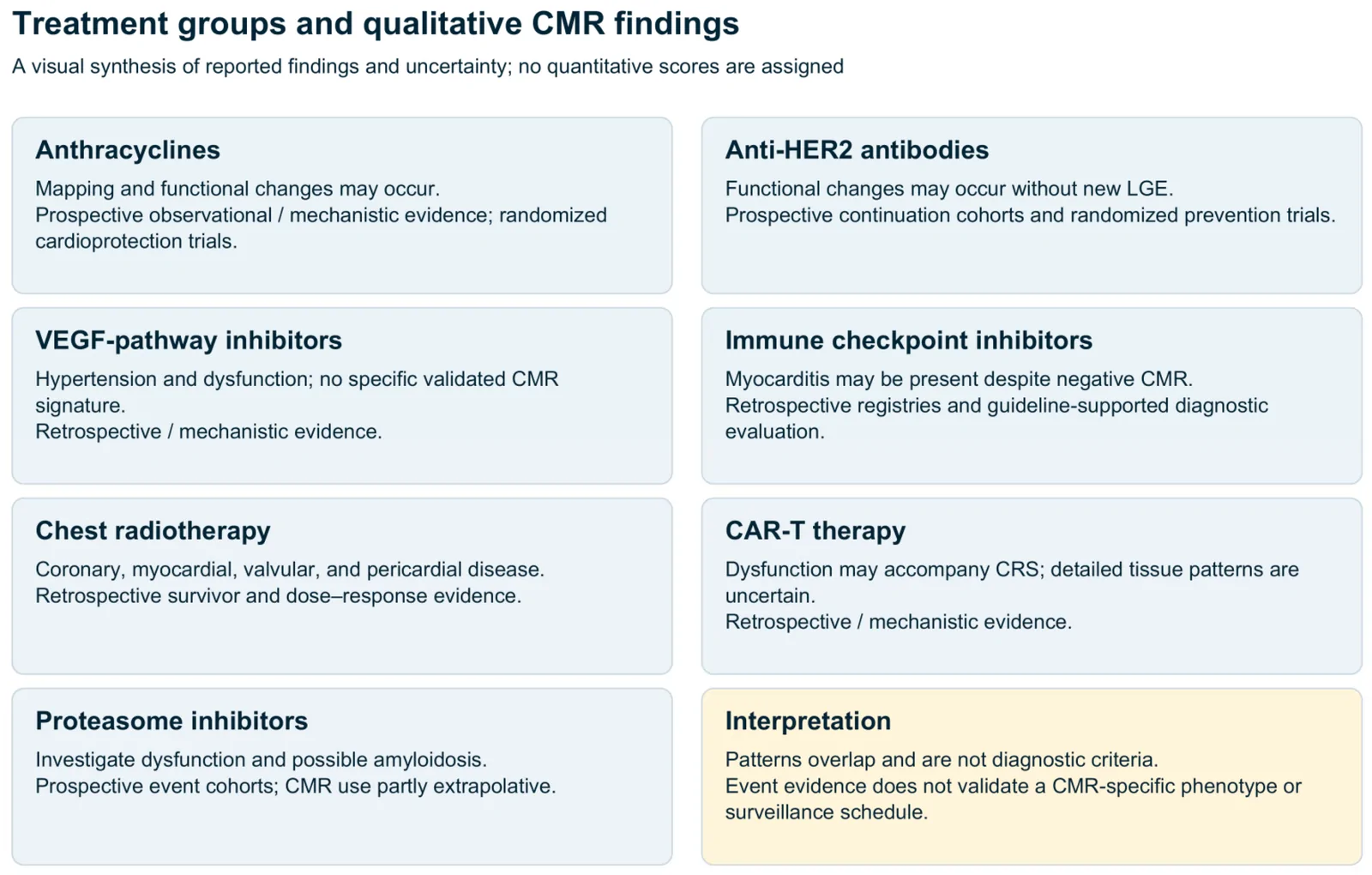
Qualitative CMR findings across seven anticancer treatment groups. The figure summarizes reported or plausible patterns and their evidence sources, not pooled effect sizes, validated diagnostic signatures, or numerical z-scores. Findings may overlap across groups. CMR-specific evidence is particularly limited for VEGF-pathway inhibitors, CAR-T therapy, and proteasome inhibitors; evidence for cardiovascular events should not be mistaken for validation of a tissue phenotype [19,20,22,23,24,27,28,43,44,49,50,51,52,53,54,55,56,57,58,59,61,62,63,64,65,66,67,68]. HER2, human epidermal growth factor receptor 2; VEGF, vascular endothelial growth factor; CRS, cytokine release syndrome.

**Figure 4:**
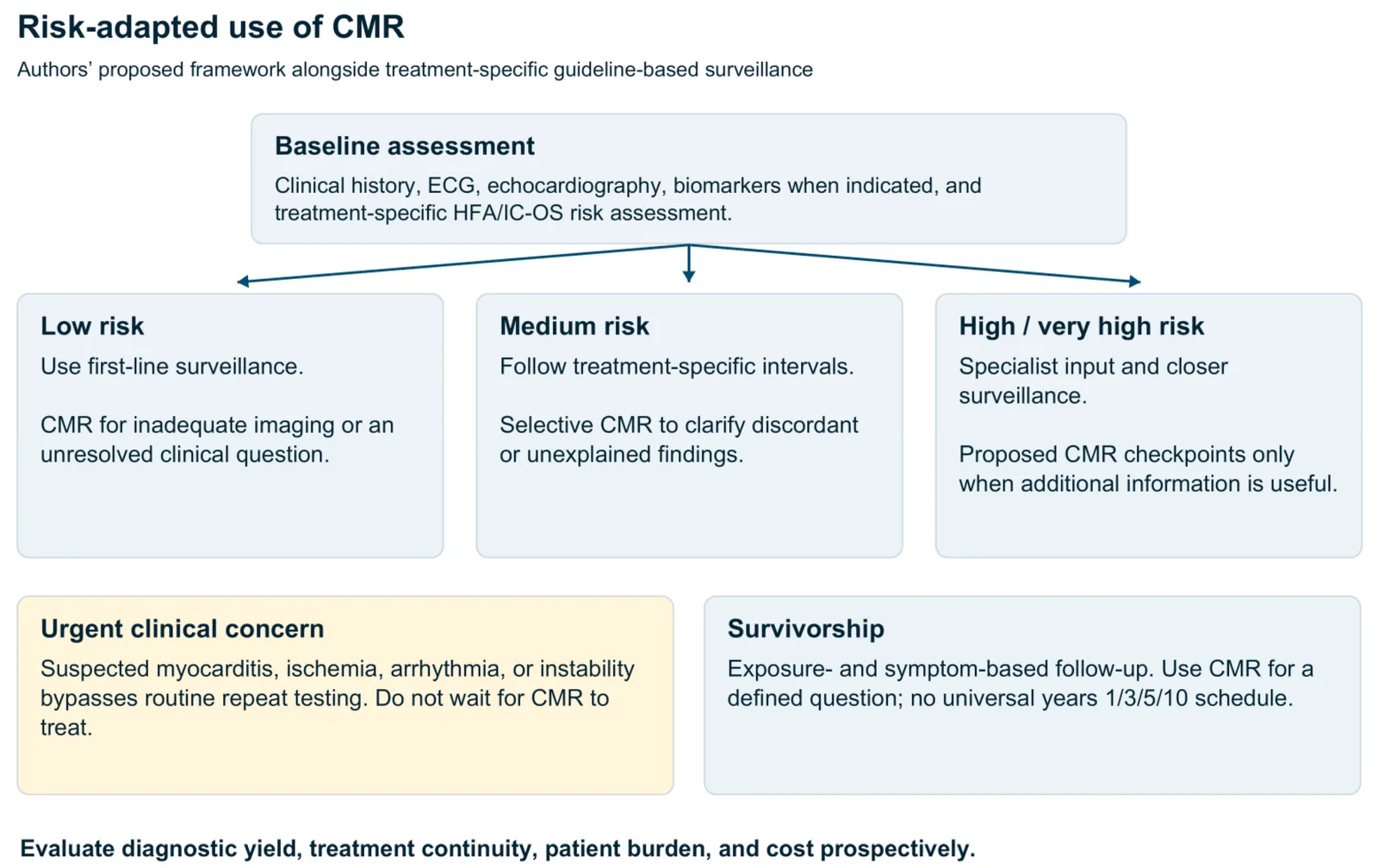
Proposed risk-adapted use of CMR alongside guideline-supported clinical assessment, echocardiography, and biomarkers [15,60,70]. Treatment-specific risk determines first-line surveillance. Proposed CMR checkpoints are optional additions for selected patients, not validated schedules. Suspected myocarditis or clinical instability bypasses routine repeat-testing pathways; investigation and treatment should not wait for CMR availability. HFA, Heart Failure Association; IC-OS, International Cardio-Oncology Society; ECG, electrocardiogram.

**Figure 5:**
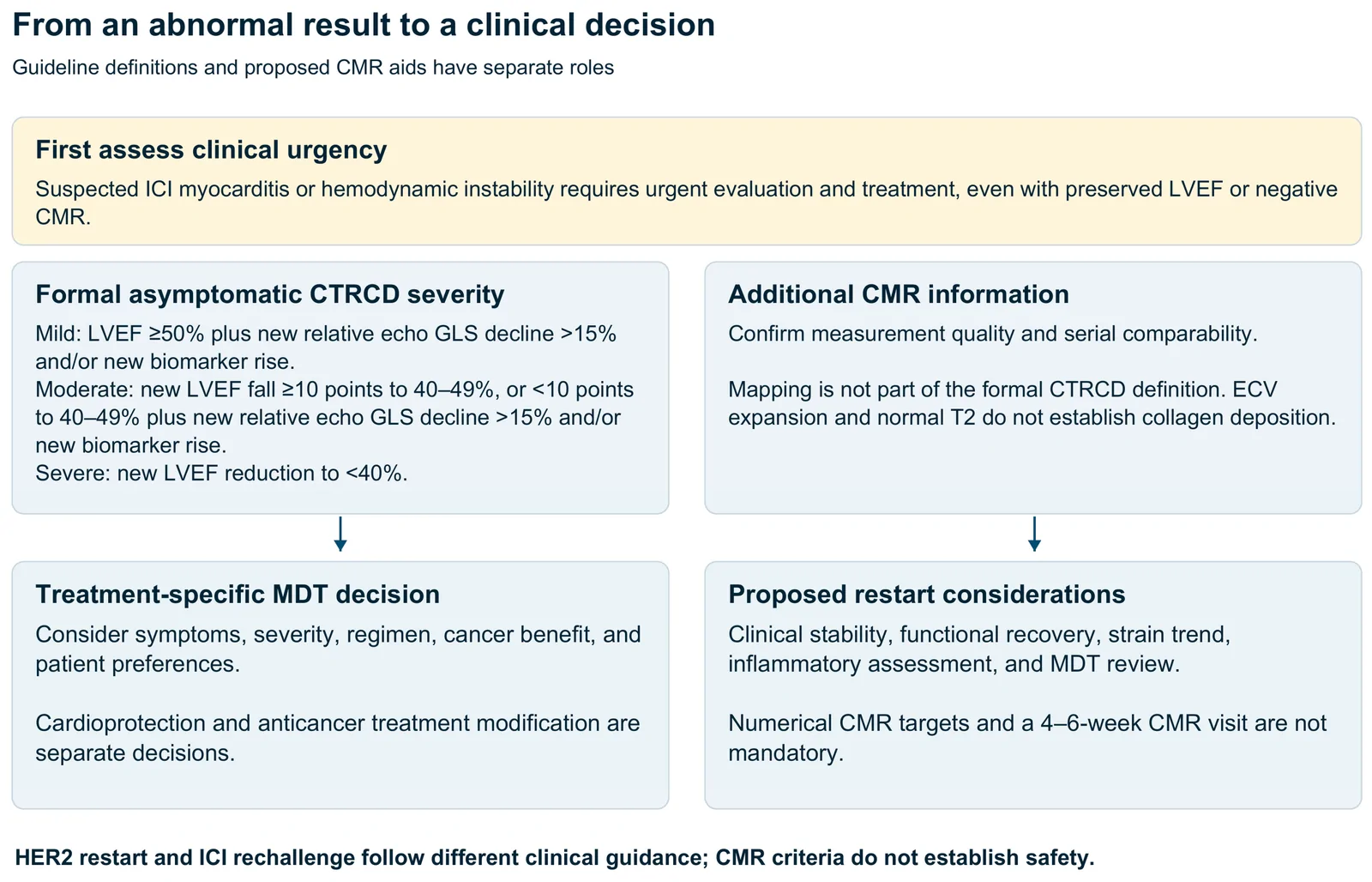
Clinical interpretation of CMR within a treatment-specific decision pathway. Formal asymptomatic CTRCD severity is based on LVEF, echocardiographic GLS, and cardiac biomarkers [15,16]. Mapping adds information but does not replace these criteria. A reproducible ECV rise is a risk signal rather than a stand-alone stopping rule, and normal T2 does not establish collagen attribution. The proposed CMR-informed restart considerations are unvalidated and subordinate to treatment-specific clinical guidance. Suspected ICI myocarditis follows an urgent clinical pathway even when LVEF is preserved or CMR is negative. MDT, multidisciplinary team; GLS, global longitudinal strain; LVEF, left ventricular ejection fraction; ECV, extracellular volume fraction.

**Table 1:** Multiparametric CMR techniques, interpretation requirements, and supporting evidence for use in CTRCD assessment.

Technique	Main Measurement	Interpretation Requirements	Contrast-Free	Evidence Supporting the Stated Use	Clinical Role and Limitation
Cine imaging	Ventricular volumes and LVEF/RVEF	Serial change interpreted against local reproducibility	Yes	Guideline-supported functional assessment [8,15]	Clarifies ventricular function when echocardiography is inconclusive; does not characterize tissue injury directly
T2 mapping	Increased tissue water	Local reference range and repeatability; z > 2 may flag an elevated value	Yes	Consensus-supported T2-based mLLC marker [9,25]; experimental and observational evidence [19,20]	Supports assessment of edema; normal T2 does not exclude inflammation or prove that ECV expansion is collagen-related
T2-STIR	T2-weighted edema signal	Image quality and validated local interpretation; conventional signal-ratio criteria are technique-dependent	Yes	Consensus-supported T2-based mLLC marker [25]	Alternative where mapping is unavailable; limited sensitivity in ICI myocarditis [23]
Native T1	Tissue changes from edema, fibrosis, or infiltration	Local reference range; serial change beyond measurement uncertainty	Yes	Consensus-supported T1-based mLLC marker [9,25]; observational evidence [20,26]	Adds tissue information without contrast; an elevated value is not etiologically specific
ECV	Extracellular expansion	A rise ≥ 3 percentage points and beyond local MDC is a proposed risk flag; measured Hct and consistent technique required	No	T1-based mLLC marker [25]; randomized substudy and observational associations [27,28,29]	Neither a fibrosis diagnosis nor a stand-alone treatment threshold; normal T2 does not establish collagen attribution
LGE	Regional contrast distribution compatible with injury or scar	Pattern, timing, and clinical context; assess competing causes	No	Consensus-supported T1-based mLLC marker [25]; retrospective ICI registry [23]	Absence does not exclude ICI myocarditis; presence does not independently establish irreversible necrosis
CMR-FT strain	Myocardial deformation	The >15% relative GLS threshold derives from echocardiographic speckle tracking. Its use in CMR-FT is extrapolative; use the same software/version and local reproducibility/MDC	Yes	Prospective observational CMR-FT evidence [30,31]; ESC/IC-OS GLS definition refers to echocardiography [15,16]	Additional functional assessment; no prospectively validated CMR-FT-specific intervention threshold
Stress perfusion/MPRI	Perfusion and perfusion reserve	Consider coronary disease, hemodynamics, and local technique	No	Mechanistic/extrapolative and limited observational support in this setting [22]	Selected assessment of ischemic or microvascular disease; no validated oncology-specific treatment cutoff

ECV = (1 − Hct) × (ΔR1_myocardium_/ΔR1_blood_); use measured Hct close to acquisition and standardized sampling conditions. MDC = 1.96 × √2 × SEM; SEM = SD × √(1 − ICC). z-scores require appropriate local reference distributions. ECV changes are expressed in absolute percentage points. The >15% relative GLS threshold belongs to echocardiographic speckle tracking; transfer to CMR-FT is extrapolative. mLLC, modified Lake Louise criteria; RVEF, right ventricular ejection fraction. Guideline/consensus support in this table concerns a stated diagnostic use, not proof that serial CMR screening improves outcomes [8,9,15,16,25].

**Table 2:** CMR findings and evidence sources by treatment group. Suggested surveillance additions are the authors’ proposed framework unless a guideline-supported diagnostic indication is specified.

Treatment Group	Examples and Indications	Reported Findings and Timing	Descriptive Evidence Source	CMR Use and Schedule Status	Decision Implication	Main Limitation
Anthracyclines	Doxorubicin, epirubicin; breast cancer, lymphoma, leukemia, sarcoma	Early mapping changes may precede dysfunction; sequence and timing vary [19,20,27,28]	Randomized evidence for selected cardioprotection [6,32,41,42]; prospective observational and mechanistic evidence for tissue changes	Proposed: selected baseline, mid-treatment, or end-of-treatment scan. Cycles 2–3 is an unvalidated translational window	Mapping changes prompt reassessment; interruption follows severity and treatment-specific guidance	ECV expansion is not collagen-specific, even with normal T2
Anti-HER2 antibodies	Trastuzumab, pertuzumab; HER2-positive breast and gastric cancer	Functional/deformation change; tissue findings are variable [20]	Prospective observational continuation studies [43,44,45]; randomized prevention trials [46,47,48]	Guideline-supported echocardiography/biomarkers; selective CMR for unresolved questions. Routine 3–4-weekly CMR is unvalidated	Selected asymptomatic patients with LVEF 40–49% may continue under specialist care [15]	Small selected cohorts; no universal CMR restart threshold
VEGF-pathway inhibitors	Sunitinib, sorafenib; RCC, HCC, GIST	Hypertension and ventricular dysfunction; no validated CMR signature [22,49]	Retrospective evidence for toxicity; mechanistic/extrapolative CMR rationale	Selected diagnostic CMR for unexplained dysfunction or suspected ischemia; no fixed surveillance schedule	Control blood pressure and assess heart failure/ischemia; treatment change depends on severity	Perfusion changes cannot by themselves distinguish direct toxicity from hypertension
ICIs	PD-1, PD-L1, CTLA-4 inhibitors; multiple cancers	Myocarditis often presents early; LGE and mapping may be negative [23,24,26]	Guideline-supported diagnostic evaluation [15]; retrospective registries [23,24,26,50]	Urgent clinically indicated CMR when feasible; no routine serial CMR screening; do not delay treatment	Interrupt for suspected myocarditis; corticosteroids when likely; MDT determines later treatment	Negative imaging does not exclude myocarditis
Chest radiotherapy	Breast, lung, esophageal, mediastinal malignancies	Coronary, myocardial, valvular, or pericardial disease may emerge years later [51,52,53,54]	Retrospective survivor and dose–response evidence; extrapolative CMR surveillance	Risk-based survivorship care; selective CMR for specific questions. Fixed years 1/3/5/10 CMR is unvalidated	Manage the identified disease and review radiation exposure with the MDT	Field-related findings do not exclude coronary disease
CAR-T therapy	Hematologic malignancies	Cardiac dysfunction often accompanies CRS; detailed CMR patterns are insufficiently defined [55,56]	Retrospective event cohorts; mechanistic/extrapolative tissue characterization	Bedside echocardiography during instability; CMR after stabilization if clinically useful	Treat CRS and provide hemodynamic/cardiac support	No established diffuse subendocardial edema signature
Proteasome inhibitors	Bortezomib, carfilzomib; multiple myeloma	Hypertension and heart failure; consider coexisting AL amyloidosis [57,58,59]	Prospective observational event data [58]; retrospective/pooled safety data [57]; extrapolative CMR use	Selective CMR for unexplained dysfunction or suspected infiltration; routine baseline screening is unvalidated	Treat the clinical syndrome; assess whether amyloidosis changes management	CMR does not establish amyloid type or drug causality alone

Evidence categories refer to the named finding or intervention, not to all entries in a row. Randomized evidence for cardioprotection does not validate the proposed CMR schedule. Guideline-supported surveillance generally uses echocardiography and biomarkers according to treatment and risk [15,60]. RCC, renal cell carcinoma; HCC, hepatocellular carcinoma; GIST, gastrointestinal stromal tumor.

**Table 3:** Treatment-specific clinical considerations. Guideline-supported management principles are distinguished from proposed CMR aids; imaging-only restart criteria have not been prospectively validated.

Treatment Group	Clinical and Imaging Assessment	Continuation or Interruption	Cardioprotective Management	Restart and Follow-Up Status
Anthracyclines	Use formal CTRCD severity [15,16]. Mapping and ECV are adjuncts; a rise ≥ 3 percentage points is not an independent stopping rule	Guideline-supported: mild asymptomatic CTRCD may continue with monitoring; moderate/severe asymptomatic or significant symptomatic toxicity generally requires interruption [15]	Treat established dysfunction as indicated. Statin prevention evidence is population-specific [32]; consider dose modification, liposomal anthracycline or dexrazoxane if restarting [15]	MDT decision. Proposed CMR recovery targets in Section 6.5 are unvalidated and not mandatory. Consider repeat CMR at 4–6 weeks only if it answers a clinical question
Anti-HER2 antibodies	Asymptomatic LVEF 40–49% is the population studied in SAFE-HEaRt [43,44]. The >15% GLS criterion is echocardiographic	Guideline-supported: selected asymptomatic moderate CTRCD may continue under specialist care; severe or significant symptomatic dysfunction warrants interruption [15]	ACEI/ARB and beta-blocker as indicated/tolerated; close clinical, echocardiographic, and biomarker follow-up	Guideline-supported: consider resumption when asymptomatic with LVEF ≥ 40%, ideally ≥50%; monitor echo/biomarkers every two cycles for the first four cycles after restart [15]. CMR targets are supplementary
VEGF-pathway inhibitors	Assess blood pressure, ischemia, symptoms, and CTRCD severity; CMR may clarify uncertain findings	Treatment-specific interruption for significant toxicity; no validated CMR-only continuation rule [15]	Optimize blood pressure and use heart-failure treatment where indicated	Individual MDT assessment after stabilization; no validated universal CMR restart criteria
ICIs	Suspected myocarditis requires urgent clinical evaluation even with normal LVEF or negative CMR	Guideline-supported immediate interruption during investigation [15]; no routine watchful-waiting pathway	When myocarditis is likely: IV methylprednisolone 500–1000 mg/day for 3–5 days; specialist escalation if refractory [15,61,62,63]	Separate individualized MDT decision after recovery assessment; proposed CMR targets cannot establish rechallenge safety
Chest radiotherapy	Investigate coronary, myocardial, valvular, and pericardial disease; consider dose distribution	Decisions concern the diagnosed cardiac condition and oncologic context; no universal CMR stopping rule	Risk-factor control and disease-specific treatment	Guideline-based survivorship follow-up; selective CMR, without a mandated years 1/3/5/10 schedule
CAR-T therapy	Assess CRS, hemodynamics, arrhythmia, and ventricular function	Urgent treatment of instability; imaging must not delay care	CRS-directed treatment and cardiac support [15,55,56]	Oncologic/cardiology review after stabilization; no validated CMR restart threshold
Proteasome inhibitors	Assess symptoms, blood pressure, dysfunction, biomarkers, and possible amyloidosis	Individualize interruption or regimen modification according to severity	Treat heart failure/hypertension and investigate infiltration where indicated [57,58,59]	MDT review of recovery and alternatives; CMR does not supply mandatory restart criteria

**Table 4:** Proposed allocation of CMR by baseline risk and clinical setting. First-line surveillance follows treatment-specific guidance; additional CMR checkpoints require individual justification [15,60,70].

Baseline Risk or Setting	Guideline-Supported First-Line Care	Proposed Additional CMR Use	Clinical Purpose
Low	Clinical assessment, echocardiography, and biomarkers as indicated by treatment	Not routine; consider for inadequate imaging or a clinically important unresolved question	Clarify findings without unnecessary testing
Medium	Treatment-specific surveillance frequency, adjusted for new findings	Selected confirmatory scan for uncertain function, suspected tissue injury, or discordance; no universal cycle or dose trigger	Resolve uncertainty that may change care
High/very high	More intensive treatment-specific surveillance and specialist input	Consider baseline or follow-up CMR where tissue or functional information is likely to be useful; cycles 2–3 remains a hypothesis for anthracyclines	Establish a reliable reference and assess evolving abnormalities
Survivorship	Follow-up based on exposure, symptoms, and end-of-treatment risk; dedicated pediatric guidance when applicable	Selected CMR for scar, perfusion, ventricular function, or inconclusive tests; no fixed universal schedule	Investigate late disease and persistent abnormalities

**Table 5:** Selected studies informing this review. Design, measured outcomes, and limits of inference are distinguished; use of a CMR endpoint does not establish benefit from CMR-guided care.

Study	Design and Population	Intervention or Measurement	Principal Result	Interpretation for This Review	Important Limitation
Cardiac CARE [6]	175 in the cohort; 57 randomized after higher hs-cTnI during anthracycline treatment	Carvedilol + candesartan vs. standard care; 6-month CMR-LVEF	Adjusted difference −0.37 percentage points (95% CI −3.59 to 2.85; *p* = 0.82); one standard-care patient developed heart failure	No demonstrated LVEF benefit for this troponin-guided strategy	Does not establish benign troponin release or superiority of CMR-guided management
Cardinale correspondence [7]	Letter and subsequent scientific discussion	Interpretation of the Cardiac CARE troponin trigger	Questions how the selected biomarker threshold identifies treatment-responsive risk	Context for cautious interpretation	Correspondence is not an independent trial or validation study
STOP-CA [32]	Double-blind randomized trial; 300 patients with lymphoma	Atorvastatin 40 mg/day vs. placebo; LVEF fall ≥ 10 percentage points to <55% at 12 months	Primary endpoint 9% vs. 22% (*p* = 0.002); heart-failure events 3% vs. 6% (*p* = 0.26)	Randomized evidence for a specific preventive intervention	Functional endpoint; no proof of benefit from CMR-guided treatment changes
STOP-CA ECV substudy [27]	171 paired CMR studies; 127 paired contrast scans suitable for ECV	Atorvastatin effect on ECV rise ≥ 3 percentage points	8% vs. 29%; odds ratio 0.20 (95% CI 0.06–0.59); greater ECV rise associated with LVEF decline	Candidate extracellular-expansion risk signal	Not histologically specific and not a validated intervention threshold
Galán-Arriola et al. [19]	Experimental serial CMR study in pigs with histological correlation	Doxorubicin exposure and early withdrawal	Week-6 isolated T2 elevation corresponded to intracellular edema; early withdrawal prevented progression	Mechanistic support for an early injury window	Human cycle 2–3 timing is unvalidated extrapolation
RESILIENCE design [69]	Published multinational phase II sham-controlled trial design	Remote ischemic conditioning and CMR-based endpoints/methods	Describes the planned study and imaging assessments	Example of a multicenter CMR research design	Design publication is not efficacy or completed feasibility evidence
SAFE-HEaRt [43,44]	Prospective single-arm study; 30 evaluable patients, asymptomatic LVEF 40–49%	HER2-targeted therapy with cardioprotective medication and close follow-up	27/30 completed planned therapy without the study-defined cardiac endpoint	Supports carefully selected continuation under specialist supervision	Small selected cohort; used echocardiography, not a mandated 3–4-weekly CMR schedule
Zhang et al. 2020 [23]	Retrospective international registry; 103 patients with ICI myocarditis and CMR	LGE and T2-weighted findings	LGE in 48%; 21.6% with CMR within 4 days of admission vs. 72.0% later	A negative LGE study does not exclude ICI myocarditis	Timing and selection affect findings; absence of LGE does not prove reversibility
Thavendiranathan et al. 2021 [26]	Retrospective international ICI myocarditis registry mapping analysis	Site-referenced T1 and T2 measurements	T1 abnormal in 78%; T2 abnormal in 43% of those with available measurements	Mapping may add evidence of injury and prognostic information	Selected cohort; not every patient had each mapping measurement; negative CMR remains possible
Wong et al. [29]	Non-oncologic clinical CMR cohort; 1176 participants	ECV in relation to mortality and heart-failure admission	Higher ECV associated with adverse outcomes	Supports general prognostic relevance of extracellular expansion	Cross-sectional ECV association does not validate serial oncology thresholds
SUCCOUR-MRI [33]	333 followed; 105 with isolated echocardiographic GLS decline > 12% randomized	Neurohormonal cardioprotection vs. usual care; CMR-LVEF endpoint	LVEF change −2.5 vs. −5.6 percentage points (*p* = 0.009); CTRCD 1/49 vs. 6/56 (*p* = 0.075)	Evidence for intervention after an echocardiographic strain signal	Does not validate CMR-FT or mapping-guided treatment thresholds
Thavendiranathan et al. [20]	Prospective cohort; 136 women receiving sequential anthracycline and trastuzumab treatment	Serial CMR tissue characterization and functional assessment	Mapping changes generally small and transient; 27% developed functional CTRCD; no new LGE	Tissue and functional abnormalities need not evolve together	Does not establish that all isolated tissue changes are benign

## Data Availability

No new data were created or analyzed in this study. Data sharing is not applicable to this article.

## Abbreviations

ACEI, angiotensin-converting enzyme inhibitor; AL, immunoglobulin light-chain amyloidosis; ARB, angiotensin receptor blocker; CAR-T, chimeric antigen receptor T-cell; CMR, cardiovascular magnetic resonance; CMR-FT, CMR feature tracking; CRS, cytokine release syndrome; CTRCD, cancer therapy-related cardiac dysfunction; ECG, electrocardiogram; ECV, extracellular volume fraction; ESC, European Society of Cardiology; GDMT, guideline-directed medical therapy; GLS, global longitudinal strain; Hct, hematocrit; HER2, human epidermal growth factor receptor 2; HFA, Heart Failure Association; GIST, gastrointestinal stromal tumor; HCC, hepatocellular carcinoma; hs-cTnI, high-sensitivity cardiac troponin I; ICC, intraclass correlation coefficient; ICI, immune checkpoint inhibitor; IC-OS, International Cardio-Oncology Society; LGE, late gadolinium enhancement; LVEF, left ventricular ejection fraction; MDC, minimal detectable change; MDT, multidisciplinary team; mLLC, modified Lake Louise criteria; MPRI, myocardial perfusion reserve index; OS, overall survival; PFS, progression-free survival; RCC, renal cell carcinoma; RDI, relative dose intensity; RVEF, right ventricular ejection fraction; SD, standard deviation; SEM, standard error of measurement; SGLT2, sodium-glucose cotransporter-2; T2-STIR, T2-weighted short-tau inversion recovery; VEGF, vascular endothelial growth factor.
